# Correction: Optimizing digital implant impressions: Evaluating the significance of scan body image deficiency and alignment under varied scan body exposures

**DOI:** 10.1371/journal.pone.0315735

**Published:** 2024-12-10

**Authors:** Pobploy Petchmedyai, Prakan Thanasrisuebwong

There is an error in the affiliation for authors Pobploy Petchmedyai and Prakan Thanasrisuebwong. The correct affiliation is: Dental Implant Centre, Faculty of Dentistry, Mahidol University, Bangkok, Thailand.
