# Supplementary material for: Optimizing digital implant impressions: Evaluating the significance of scan body image deficiency and alignment under varied scan body exposures
Source: PLoS One. 2023 Sep 21;18(9):e0291831. doi: 10.1371/journal.pone.0291831 (PMC10513296; doi:10.1371/journal.pone.0291831)
Supplement: S1 File — (DOCX) [file pone.0291831.s001.docx]

Software used for generating virtual scan body image deficiency in this study is CAD software (Meshmixer 2017 version 3.5.474, Autodesk, USA). The process of measurement are as follows:

1. Importation of STL File:
   - Begin by importing the STL file of the 3D implant model containing the scan body.
2. Utilization of "Plane Cut" Function:
   - Navigate to the "Edit" menu and select the "Plane Cut" function.
   - Apply this function to remove a portion of the scan body, such as the upper 1/4 or both the upper and lower 1/4 parts.
   - To ensure precise alignment of the cut plane with the cylinder's center, enable the "Show Local Axis" option in the toolbar. This feature displays the local axis of the cut plane.
   - Adjust the position and orientation of the cut plane using the rotation and translation handles until the local axis aligns with the cylinder's center axis.
   - Additionally, the "Snap to Grid" option in the toolbar can be employed to align the cut plane more accurately with the grid or other reference points.
3. Saving the Modified STL Files:
   - After removing the desired portion of the scan body, save the new STL files.
   - These modified files, reflecting the scan body image deficiency, are now ready for subsequent 3D measurements.

The 3D analysis software used for 3D measurements in this study is GOM Inspect software version 2019, developed by GOM in Germany. The process for conducting the measurements is outlined as follows:

1. Creation of a New Project:
   - Begin by creating a new project within the software.
2. Importation of STL Files:
   - Import the STL file of the tested 3D model, treating it as a "CAD body."
   - Import the STL file of the reference 3D model, considering it as a "Mesh."
3. Initial Alignment:
   - In the operation section, utilize the "pre-alignment" function during the initial alignment process. This function ensures that the tested and reference STL files are aligned within the same coordinate system.
4. Angulation Deviation Measurement:
   - To measure the angulation deviation of implant positions, generate a cylinder at the virtual implant locations.
   - Calculate the angular deviation by comparing the cylindrical axes of the virtual implants.
5. Linear Implant Deviation Measurement:
   - Measure the linear deviation of implant positions by identifying two intersection points.
   - Construct these points using the cylindrical axis and the top plane of the implants.
