# Supplementary material for: Optimizing digital implant impressions: Evaluating the significance of scan body image deficiency and alignment under varied scan body exposures
Source: PLoS One. 2023 Sep 21;18(9):e0291831. doi: 10.1371/journal.pone.0291831 (PMC10513296; doi:10.1371/journal.pone.0291831)
Supplement: Attachment — Submitted filename: Response to reviewer 2 comment.docx [file pone.0291831.s003.docx]

**Response to reviewer 2 comments:**

We would like to express our gratitude for your valuable feedback on our manuscript. We sincerely appreciate their time and effort in providing a thorough evaluation. Your comments have helped us identify the areas that require revision and improvement. In the following sections, we address each of your points separated in 3 sections: Comment, Response, Text change. Highlight the revisions made in grey color for your convenience. It's worth noting that beyond the revisions you suggested, we have also performed further edits that we believed necessary to improve the overall quality and clarity of the manuscript. These changes, performed for the enhancement of our manuscript, were tracked in red in the revised with track change manuscript file.

**COMMENT:** It needs to rewrite the whole abstract. Most of the abstract about introduction and nothing about the actual study. For example “This study provides valuable insights into the factors that could affect the accuracy of virtual implant positions obtained from digital impressions. “How ? nothing mentioned.

**RESPONSE:** we acknowledge the need to provide a clearer description of the actual study and the insights gained from it. We have conducted a comprehensive revision of the abstract, incorporating precise elaboration within the material and methods as well as results sections.

**TEXT CHANGE:** Please find the rewritten abstract as follow.

“In implant dentistry, the advent of intraoral scanning technology has revolutionized traditional clinical processes by streamlining procedures and ensuring predictable treatment outcomes. However, achieving accurate virtual implant positions using intraoral scanners and scan bodies can be influenced by various clinical and laboratory factors. This study aims to investigate the impact of scan body image capture deficiency and scan body alignment methods in computer-aided design (CAD) software on the accuracy of virtual implant positions, particularly in different implant depths.

Three stereolithographic half-arch implant models with different implant depths were prepared, representing three scenarios of scan body exposure: full exposed scan body, 2/3 exposed scan body, and 1/3 exposed scan body. The scan body image capture deficiency and alignment methods were simulated using CAD software. The deviation of virtual implant positions obtained from different scenarios were evaluated using 3D analysis software. The highest angular and linear deviation (0.237±0.059 degrees, 0.084±0.068 mm) were found in the 1/4 upper and lower part scan body deficiency using the 1-point alignment method in the 1/3 exposed scan body. Two-way ANOVA analysis revealed significant effects of scan deficiency on virtual implant position deviations across all scan body exposures, except for the linear deviation when the scan body was exposed 2/3 of its length. Furthermore, scan deficiencies in the 1/4 upper and lower parts of the scan body significantly affected implant angular deviation regardless of scan body exposure, while implant linear deviation was specifically affected when the scan body was exposed to only 1/3 of its total length.

Deficiencies in scan body acquisition, particularly in deep soft tissue situations, can lead to deviations in both angular and linear positioning of virtual implants. Employing appropriate scan body alignment methods such as a 3-point alignment approach demonstrates better accuracy compared to a 1-point alignment.”

**COMMENT:** There is no 0.000 P value. Change it to <0.001

**RESPONSE:** Regarding the mentioned *P* value, we apologize for the error and appreciate for pointing it out. We have corrected it to "<0.001" to accurately reflect the statistical significance.

**-COMMENT:** Who did the scan ? all of them by the same person ? did he/she has enough experience to use it ? who did the defect ? ….etc

**RESPONSE:** We appreciate your comment and understand the importance of clarifying the details regarding the scan procedure and operator experience. In this study, all scans were performed by a single experienced operator who had sufficient expertise in using intraoral scanners and CAD software. The operator had undergone training and had prior experience in conducting scans and measurements in 3D analysis software. This approach ensures consistency and reproducibility in the scanning process. To create the scan body deficiencies, the operator followed a standardized method in the CAD software, as described in the Methods section. The steps for generating scan body deficiencies were carefully executed by the experienced operator to ensure reproducibility of the results. By having a single operator perform all the scans and manipulations, potential variations or biases introduced by multiple operators were minimized, enhancing the reliability of the study.

**TEXT CHANGE:** We have included more detail regarding the mentioned issue in the last paragraph of Material and Method section as follows.

“In this study, all scans were performed by a single experienced operator who had sufficient expertise in using intraoral scanners and CAD software. The operator had undergone training and had prior experience in conducting scans, using CAD software and measurements in 3D analysis software.”

“To ensure reproducibility, a standardized method in the CAD software to create scan body deficiencies was followed. The method involved utilizing the “plane cutting” technique to remove specific portions of the scan body, aligning the cut plane with the cylinder's center axis, the “Snap to Grid” was employed to improve alignment accuracy with reference points and saving the modified STL files for subsequent 3D measurements.” ( Highlighted in yellow)

**COMMENT:** *P* value should capital and italic

**RESPONSE:** Thank you for pointing out the error in the formatting of the *P* value. We apologize for the oversight. In the revised manuscript, we have corrected the formatting to ensure that the *P* value is capitalized and italicized throughout the document.

**COMMENT:** What are the limitations of this study ?

**RESPONSE:** We acknowledge the need to address the limitations of our study. Several limitations should be considered when interpreting the findings.

**TEXT CHANGE:** The following were included in the last paragraph in the discussion part.

“Despite the valuable findings, there are certain limitations that should be acknowledged. Firstly, the study utilized simulated implant models, which may not fully replicate the complexity and variability of clinical scenarios. As mentioned earlier, further research using actual patient cases is needed to validate these results. Secondly, the study focused on specific CAD software and scan body alignment techniques. The findings may not be directly applicable to other software or alignment methods, and therefore, caution should be exercised when generalizing the results to different systems. Additionally, the study evaluated the accuracy of virtual implant positions but did not assess the clinical outcomes or long-term success of the restorations based on these positions. Further investigations involving clinical studies are required to determine the impact of scan body image capture deficiency and alignment methods on the clinical efficiency as well as overall short- and long-term treatment outcomes. Lastly, the sample size in this study was relatively small, which may limit the generalizability of the results. A larger sample size would provide more robust statistical analyses and a better understanding of the relationship between scan body deficiencies, implant depth, and virtual implant accuracy.”.
