# Supplementary material for: Optimizing digital implant impressions: Evaluating the significance of scan body image deficiency and alignment under varied scan body exposures
Source: PLoS One. 2023 Sep 21;18(9):e0291831. doi: 10.1371/journal.pone.0291831 (PMC10513296; doi:10.1371/journal.pone.0291831)
Supplement: Attachment — Submitted filename: Response to reviewer 4.docx [file pone.0291831.s004.docx]

**Response to reviewer 4 :**

Thank you for your valuable feedback regarding additional comparison with other similar research findings and the clarity of the images in our manuscript. We sincerely appreciate your comments, and we have taken them into consideration during the revision process.

During the literature review process, we found that only one study had specifically assessed the impact of scan body deficiency on the accuracy of virtual implant positioning in CAD software. This study, conducted by Park in 2020, utilized a different scanning system and provided valuable insights into the adverse consequences of inadequacies in scanned images of a scan body on accuracy of virtual implant position. The scarcity of research focusing on this particular aspect highlights the novelty and significance of our study in contributing additional evidence and further understanding the critical importance of addressing scan body image deficiencies for precise virtual implant position.

A comparison with this research has already been included in the Discussion section of our manuscript as below.

“These findings are in agreement with a previous study [13], which utilized a different scanning system and also demonstrated the adverse impact of inadequacies in scanned images of a scan body on implant positioning accuracy in CAD software. Similar to our study, the previous research identified increasing deviations with higher deficiency levels. The extent of the deviations in the previous study, reaching up to 0.081.6 ± 0.002 mm in linear discrepancy and 0.26 ± 0.01 degrees in angular discrepancy at 15% of scan body deficiency. While in our investigation, we introduced an additional variable of implant depth, and the highest deviations at 0.084 ± 0.068 mm and 0.237 ± 0.059 degrees, were observed when the implant was placed at a greater depth, only 1/3 of scan body exposed, and with a scan deficiency in the 1/4 upper and lower part of the scan body. These congruent results underscore the critical importance of addressing scan body image deficiencies to ensure precise virtual implant positioning. The fact that both studies, despite using different scanning systems and slightly different experimental setups, produced comparable results regarding the impact of scan deficiency on implant accuracy further validates the significance of these observations.”

Regarding the clarity of the images, we are pleased to inform you that we have made significant improvements to all the figures in accordance with the journal's requirements. To achieve this, we utilized the Preflight Analysis and Conversion Engine (PACE) digital diagnostic tool, as suggested by the journal. This tool ensured that all our figure files now meet the specified guidelines. We followed the provided instructions on the PACE platform, which allowed us to make the necessary adjustments and modifications to each figure file.

All figure files are now in TIFF and the dimensions of the figures are within the specified range, with a width between 789 and 2250 pixels (at 300 dpi) and a maximum height of 2625 pixels (at 300 dpi). The resolution of each figure is now set at 300 to 600 dpi, and we have successfully reduced the file sizes to be under 10 MB. as per the journal's instructions. We believe that these modifications have significantly enhanced the clarity and quality of the figures in our manuscript, making them more suitable for publication.

Once again, we express our gratitude for your thorough evaluation and constructive feedback, which has undoubtedly improved the quality of our work. We look forward to any further comments or suggestions you may have during the review process.
